# Supplementary figures and images for: Identification of Sulfate Transporter Genes in Broussonetia papyrifera and Analysis of Their Functions in Regulating Selenium Metabolism
Source: Plants (Basel). 2025 Sep 27;14(19):2995. doi: 10.3390/plants14192995 (PMC12525679; doi:10.3390/plants14192995)

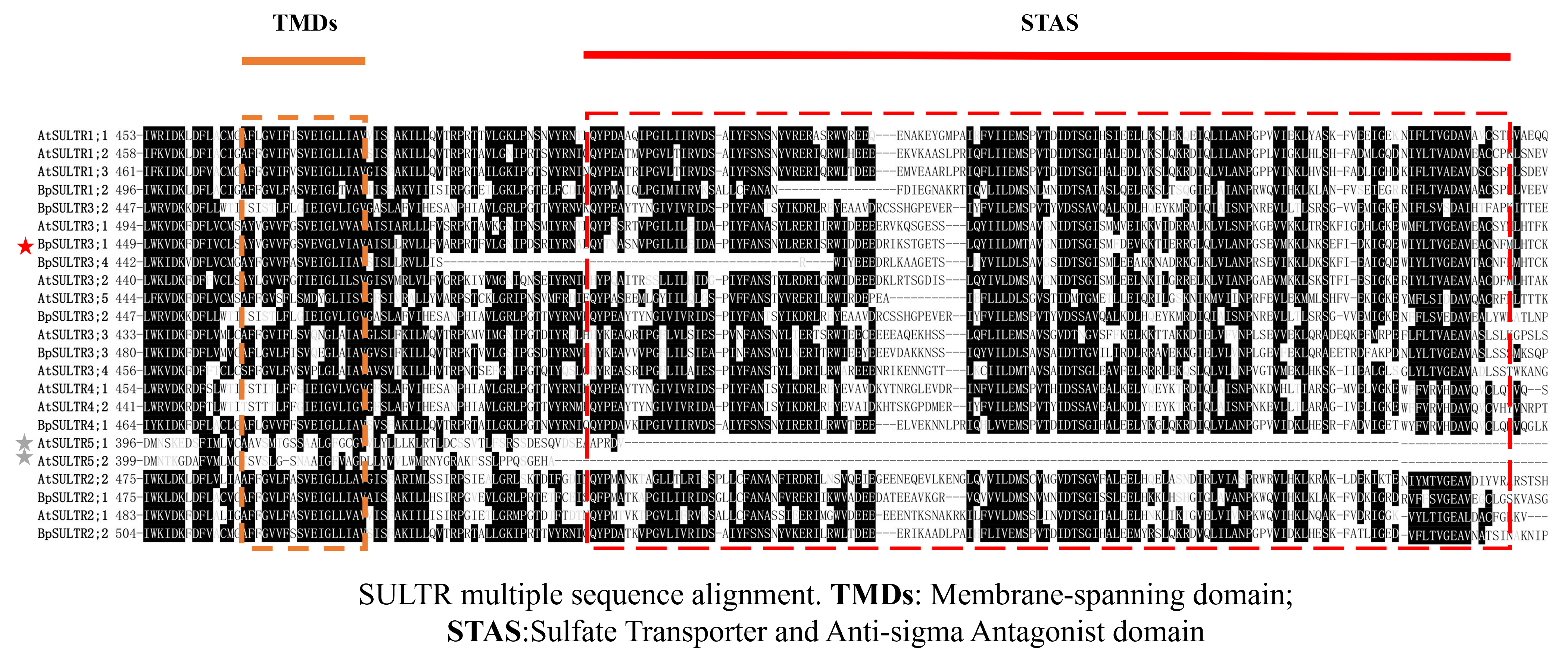

Supplement: Supplementary file 1 [file plants-14-02995-s001.zip › Figure1 multiple sequence alignment.tif]
